# Diverse Systems for Efficient Sequence Insertion and Replacement in Precise Plant Genome Editing

**DOI:** 10.34133/2020/8659064

**Published:** 2020-07-28

**Authors:** Yingxiao Zhang, Yiping Qi

**Affiliations:** ^1^Department of Plant Science and Landscape Architecture, University of Maryland, College Park, Maryland 20742, USA; ^2^Institute for Bioscience and Biotechnology Research, University of Maryland, Rockville, Maryland 20850, USA

## Abstract

CRISPR-mediated genome editing has been widely applied in plants to make uncomplicated genomic modifications including gene knockout and base changes. However, the introduction of many genetic variants related to valuable agronomic traits requires complex and precise DNA changes. Different CRISPR systems have been developed to achieve efficient sequence insertion and replacement but with limited success. A recent study has significantly improved NHEJ- and HDR-mediated sequence insertion and replacement using chemically modified donor templates. Together with other newly developed precise editing systems, such as prime editing and CRISPR-associated transposases, these technologies will provide new avenues to further the plant genome editing field.

## 1. Main Text

**Figure 1:**
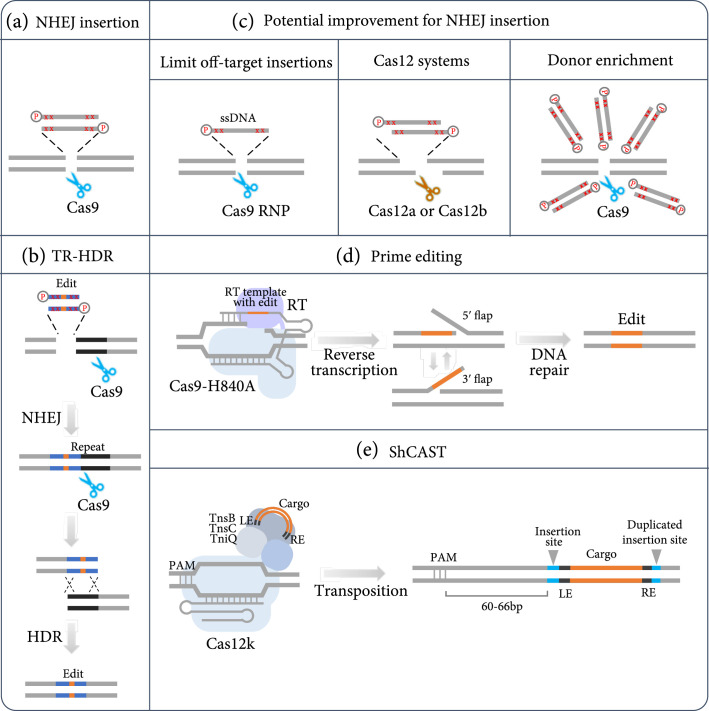
Schematic illustration of targeted sequence insertion and replacement technologies. (a) NHEJ-mediated targeted sequence insertion using chemically modified DNA donor templates. (b) Targeted sequence replacement using the tandem repeat-HDR strategy (TR-HDR). (c) Potential improvement for NHEJ-mediated sequence insertion, including using ssDNA or RNP to minimize off-target insertions, using the Cas12a or Cas12b system to induce staggered DSBs, and localized donor enrichment. In (a–c), “P” represents 5${}^{\prime }$-phosphorylation and red “x” indicates phosphorothioate linkage. (d) Precise genome editing by primer editing. RT: reverse transcriptase. (e) Large sequence insertion using ShCAST. LE: transposon left end; RE: transposon right end.

Clustered Regularly Interspaced Short Palindromic Repeats- (CRISPR-) mediated genome editing is a powerful and versatile tool for manipulating nucleic acids. However, targeted sequence insertion and replacement remains a significant challenge in plants. The efficiency of homology-directed repair (HDR) is usually low and inconsistent; as in plant somatic tissues, the predominant pathway to repair double-strand breaks (DSBs) is nonhomologous end joining (NHEJ). Many attempts have been made to improve HDR efficiencies, including actively selecting desired sequences (such as selective markers) [1–3], manipulating repair pathways [4], enriching donor templates near Cas nucleases [5], and increasing the amount of donor templates by simply providing more template or by using novel strategies such as geminivirus replication [6–8] and RNA transcription [9]. NHEJ-mediated gene insertion has also been demonstrated in plants with low editing efficiencies [10]. To develop a robust and efficient method to achieve targeted sequence insertion and replacement in plants, Lu et al. has improved the NHEJ-mediated DNA insertion approach using chemically modified donor DNA (Figure 1(a)) [11]. Furthermore, this approach has been leveraged to develop a tandem repeat-facilitated HDR strategy (TR-HDR) (Figure 1(b)) [11]. This study significantly increased the efficiencies of NHEJ- and HDR-mediated DNA insertion and replacement when compared to previous studies. The demonstrated methodology should be applicable in other plants and enable precise genome editing in basic plant research and crop breeding.

Lu et al. first focused on NHEJ-mediated gene insertion. To improve the double-stranded DNA (dsDNA) donor stability, two phosphorothioate linkages were added at the 5${}^{\prime }$ and 3${}^{\prime }$ ends of both DNA strands. In addition, 5${}^{\prime }$-phosphorylation has been used to facilitate the NHEJ repair [11, 12]. Significantly higher insertion efficiencies were observed using modified dsDNA compared to unmodified dsDNA and single-stranded DNA (ssDNA). When targeting multiple genes separately or simultaneously using short (<70 bp) modified dsDNA donors, high efficiencies (10.6%-47.3%) were observed, suggesting this approach is highly robust and efficient. Furthermore, longer donors with lengths of 526 bp and 2,049 bp were simultaneously inserted into two loci, with 25.5% and 10.5% combined (two loci) insertion frequencies, respectively, indicating this approach is capable of inserting long DNA fragments.

Lu et al. further applied this approach to improve HDR efficiency. When tandemly repeated sequences are present near DSBs, higher HDR efficiencies have been observed. This is likely due to the repeat sequences being used as a repair template, based on the synthesis-dependent strand annealing (SDSA) mechanism [13]. Therefore, Lu et al. developed a tandem repeat-HDR strategy (TR-HDR) to achieve targeted sequence replacement. A repeat sequence with desired edits is inserted to serve as a template, using the firstly established insertion strategy. At the same time, a target site for the same guide RNA (gRNA) is formed between the two repeats, thus inducing the DSB followed by HDR. This method was successfully used to introduce base substitutions and in-locus tags. The precise editing efficiencies ranged from 3.4 to 11.4%. TR-HDR provided a novel avenue to obtain robust sequence replacement and insertion through HDR.

Collectively, this study made a breakthrough for targeted sequence insertion and replacement in plants that are amenable to biolistic delivery. High editing efficiencies were achieved through NHEJ (25% on average) and TR-HDR (6.1% on average), without actively selecting edited sequences, allowing complex, precise editing to be efficiently achieved at any desired genomic locus. This simple and reliable strategy was demonstrated to introduce gene regulatory elements, protein tags, and multiple base changes. It could be further applied for gene insertion and replacement, multiplexed single nucleotide polymorphism (SNP) introduction, promoter engineering, etc.

It is notable that this technology can be further improved by overcoming some limitations discussed in this study. The first concern is the random insertion of the repair template and CRISPR reagents into the plant genome, which is also the common problem caused by biolistic delivery. Although this issue can be resolved by generating large $T_{0}$ populations followed by selection, it would be time- and labor-consuming in practice. Using chemically modified ssDNA as the donor is another option to minimize off-target insertions (Figure 1(c)). Although unmodified ssDNA performed poorly in this study, editing efficiency could be significantly increased when using chemically modified templates. In addition, ribonucleoprotein (RNP) delivery of CRISPR reagents has been successfully used for plant genome editing to achieve transgene-free genome editing and limit off-target effects [14, 15] (Figure 1(c)). However, RNP-mediated sequence insertion and replacement is still a substantial challenge in plants. The second concern is the chimerism in $T_{0}$ plants, likely because edits happened at late stages of the transgenic plant regeneration. One possible solution is to further improve the editing efficiency. Localized donor enrichment through protein-protein or protein-DNA interactions can be considered [5, 16–19] (Figure 1(c)). Moreover, Cas12-induced DSBs with staggered ends could potentially facilitate donor insertion (Figure 1(c)).

Recently, CRISPR technologies for sequence insertion and replacement have been advanced rapidly. Prime editing utilizes a reverse transcriptase fused to a Cas9-H840 nickase and a prime editing guide RNA (pegRNA) that encodes the target sequence and the desired edit, to achieve substitutions, deletions, and up to 44 bp insertions [20] (Figure 1(d)). Since prime editing enables flexible genome edits without introducing DSBs and donor templates, it has been applied in plants albeit with low efficiencies in many cases [21–26]. To achieve large sequence insertion, a CRISPR-associated transposase from cyanobacteria *Scytonema hofmanni* (ShCAST) has been characterized [27] (Figure 1(e)). ShCAST, consisting of Cas12k and three Tn7-like transposase subunits, is able to integrate DNA into specific *Escherichia coli* genomic sites 60 to 66 bp downstream of the protospacer adjacent motif (PAM) with up to 80% efficiency [27]. Although this type of DNA insertion is not seamless, it can still be used to insert sequences to introns, around regulatory elements, and in safe harbor loci. However, this system has not been demonstrated in any other organisms. These technologies, along with the one developed by Lu et al., will expand the scope and capabilities of precise and complex genome editing in plants, leading to more biotechnological and agricultural applications.
